# Chronic ventricular lead perforation: Expect the unexpected

**DOI:** 10.1002/ccr3.2005

**Published:** 2019-01-29

**Authors:** Baldeep S. Sidhu, Ronak Rajani, Christopher A. Rinaldi

**Affiliations:** ^1^ Cardiology Department Guy’s & St Thomas’ Hospitals London UK; ^2^ Division of Imaging Sciences and Biomedical Engineering King's College London London UK

**Keywords:** cardiac computer tomography, chronic pacemaker lead perforation, pacemaker complication, transvenous lead extraction

## Abstract

A high index of suspicion is needed to diagnose a chronic right ventricular lead perforation. They should be suspected in patients who develop breathlessness and have a sudden change in pacing parameters. Contrast‐enhanced CT provides high diagnostic accuracy. They can often be extracted percutaneously and rarely require surgical intervention.

## 1 INTRODUCTION

Cardiac perforations are a rare but life‐threatening complication of pacemaker implantations. Acute perforations are well described but chronic perforations are much less common and can be difficult to diagnose. We present a case report of a patient that developed a chronic right ventricular (RV) lead perforation fourteen years after initial implantation. This was an unexpected finding as imaging had been performed to look for perforation of a newly implanted RV lead. The perforated lead was extracted percutaneously without any complication. Given the rarity of chronic RV lead perforations, clinicians must understand the potential signs which may point toward this diagnosis.

## 2 CASE REPORT

A 61‐year‐old female had undergone a dual chamber pacemaker for high‐degree atrioventricular block with exertional dyspnoea in 2004 with a dual chamber pacemaker incorporating an active fixation lead to the RV apex (Guidant 4064) and a passive lead to the right atrial appendage (Guidant 4097).

**Figure 1:**
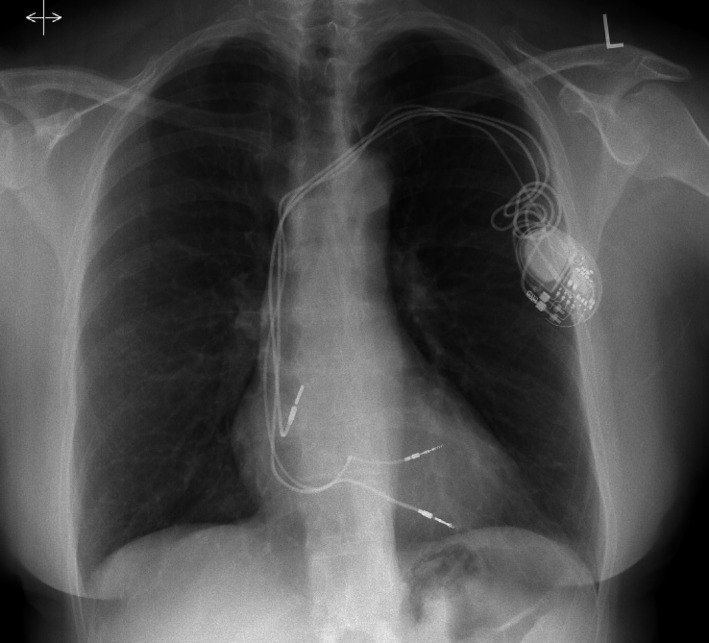
Chest X‐ray showing a dual chamber pacemaker with a redundant right ventricular (RV) lead to the RV apex, RV lead to the right ventricular outflow tract and a right atrial lead to the right atrial appendage. The chronic RV lead does not appear to be outside the cardiac silhouette

**Figure 2:**
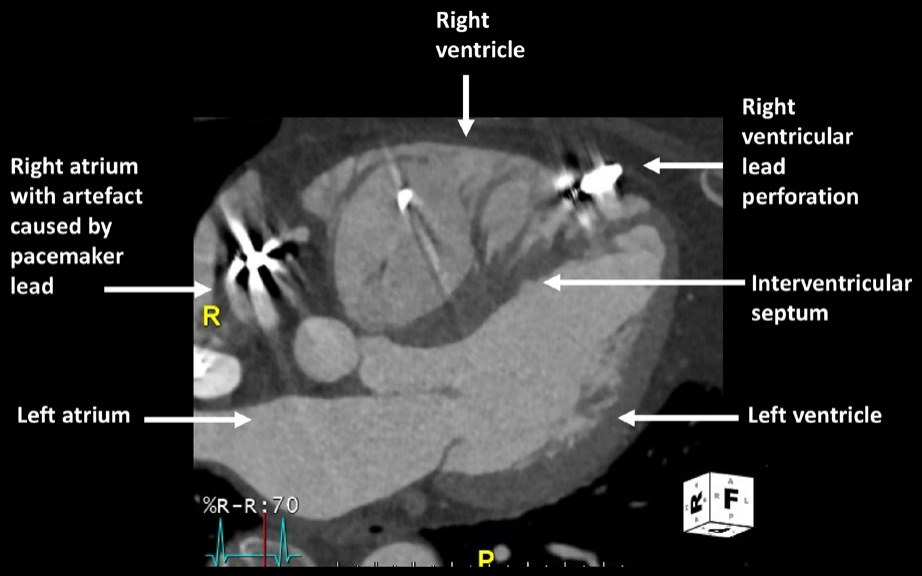
ECG‐gated contrast‐enhanced cardiac CT showing the apically sited chronic right ventricular lead perforating through the right ventricle

**Figure 3:**
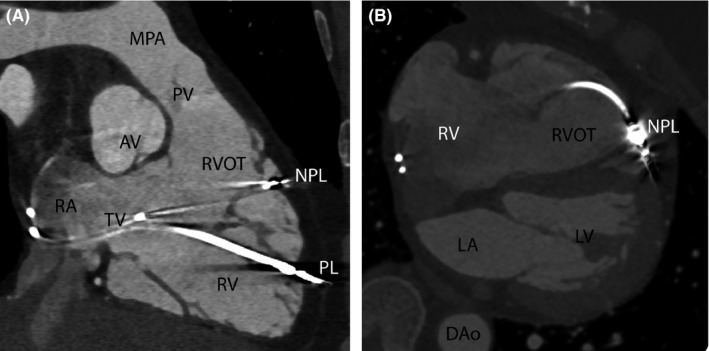
ECG‐gated contrast‐enhanced CT. A, shows perforation of one of the pacing leads (PL) through the right ventricular myocardium and visceral pericardium. B, shows the new pacing within the right ventricular outflow tract with no evidence of myocardial perforation. AV, aortic valve; Dao, descending aorta; LA, left atrium; LV, left ventricle; MPA, main pulmonary artery; NPL, nonperforated lead; PL, perforated lead; PV, pulmonary valve; RA, right atrium; RV, right ventricle; RVOT, right ventricular outflow tract; TV, tricuspid valve

Two months prior to her presentation, she had received a new RV lead, placed to the right ventricular outflow tract (RVOT; Medtronic Capsurefix Novus 5076) due to an increase in her chronic RV lead threshold (1.75 V at 1 ms) and impedance value (increased from 650 ohms six months previously to 740 ohms). The postprocedural chest X‐ray (CXR) and echocardiogram showed normal positioning of the pacing leads with no complication. She subsequently complained of intermittent chest pains and dyspnoea and was referred to our institution for further investigation. A CXR (Figure 1), echocardiogram and pacemaker checks were satisfactory with the new RV lead having a stable threshold (1 V at 1 ms) and impedance value (570 ohms) since implantation. Despite this, given her clinical presentation and unexplained symptoms, the suspicion remained that the RVOT lead may have perforated through the myocardium. To investigate this further, a contrast‐enhanced ECG‐gated cardiac computed tomographic (CT) scan was performed. This showed normal siting of the new RVOT lead but the unexpected finding of myocardial perforation as a result of the chronic RV apical lead (14 years old lead; Figures 2 and 3). The case was discussed at a multi‐disciplinary meeting, and a consensus reached for the perforated lead to be extracted. Following a discussion with the patient, it was decided to extract both her atrial and RVOT lead to enable the implantation of a fully MRI compatible device. The newly sited RV lead was removed with traction alone but a 14 Fr laser sheath was needed to extract both the chronically implanted RV and atrial lead in a hybrid lab without complication. A new dual chamber system was placed, and the patient was discharged home. Upon review in clinic, her symptoms had completely resolved.

## 3 DISCUSSION

In the current case, we report the unsuspected finding of a RV perforation from a historical pacing lead, in a patient who presented soon after new RV lead implantation. Our report illustrates a number of important learning points. Firstly, it is crucial to maintain a high index of suspicion regarding the possibility of a lead perforation in patients presenting with atypical symptoms. Secondly, as in our case, it is important not to assume that any perforation may the sequelae of a recent intervention. Although perforation was suspected, this was felt to be most likely due to the newly implanted lead when in fact it was related to the chronically implanted lead (14 years. old), which had been functioning satisfactorily for many years. In retrospect, the change in capture threshold and impedance observed during device follow up likely heralded the perforation although it is interesting to note chest pains only became prominent after implantation of the new lead. To our knowledge, this is the first case to document a chronic RV lead perforation fourteen years after its initial implantation. Finally, our case illustrates not only the use of cardiac CT in detecting lead perforations when other checks remained unremarkable, but also the successful percutaneous removal of a chronic lead perforation 14 years after implantation.

Complications arising from pacemaker lead perforations are well described in the literature and can be life‐threatening.^1^ Perforations often present acutely but delayed presentations have also been reported.^1^, ^2^ In a retrospective study of 3822 pacemaker leads, 76% of all perforations occurred within 24 hours of initial pacemaker implantation and in total only 0.8% of patients suffered from clinically significant cardiac perforations.^3^ In addition, they showed that an apically placed RV lead and female sex were both independent predictors of cardiac perforations. Chronic lead perforations are rare but several important clues may help point toward the diagnosis. Patients can present with a range of symptoms but typically complain of sudden onset chest pain or breathlessness.^4^, ^5^ They often develop altered electrical parameters on pacing checks,^2^ and these tend to be a sudden change rather than gradual deterioration which may instead indicate a malfunctioning lead. Furthermore, altered pacing parameters can occur in asymptomatic cardiac lead perforations. Therefore, there were several clues in this patient's history which may have resulted in an earlier diagnosis and the potential to avoid unnecessary interventions.

Pacemaker lead perforations can be reliably diagnosed with CT scans or echocardiography.^1^, ^6^, ^7^, ^8^ CT scanning has the added benefit of providing excellent detail and reconstruction to help guide further interventions. Adjacent viscera can also be inspected for any potential damage which makes this a useful imaging modality.

The management of chronic RV lead perforations can be difficult and there is a need to balance the risks of transvenous lead extraction (TLE) against open extraction.^1^, ^9^, ^10^ Studies suggest that TLE has good outcomes and open extraction is rarely required.^2^, ^10^ In our experience, chronically perforated leads can frequently be removed using simple extraction techniques and if these fail, then laser‐assisted extraction is almost always successful.^7^

## 4 CONCLUSIONS

Chronic RV lead perforations are rare but should be considered in patients that present with atypical symptoms and a sudden change in electrical parameters during pacing checks. Where pacing lead perforations are suspected, these should be ideally confirmed with gated cardiac CT which provides a high diagnostic accuracy in defining the culprit lead. In the current case, perforation was suspected but not in the lead in which it was found. As Heraclitus said, “If you do not expect the unexpected you will not find it, for it is not to be reached by search or trail”.

## CONFLICT OF INTEREST

None declared.

## AUTHOR CONTRIBUTION

BSS: produced the manuscript, edited Figures, provided revisions and liaised with the publisher. RR: produced the images used in the Figures, helped edit the manuscript and provided revisions on different versions of the manuscript. CAR: helped produce the manuscript and provided revisions on different versions of the manuscript.
